# Supplementary material for: Predicting Functions of Uncharacterized Human Proteins: From Canonical to Proteoforms
Source: Genes (Basel). 2020 Jun 21;11(6):677. doi: 10.3390/genes11060677 (PMC7350264; doi:10.3390/genes11060677)
Supplement: Supplementary file 1 [file genes-11-00677-s001.zip › SUPPLEMENTARY/Figure S5.pdf]

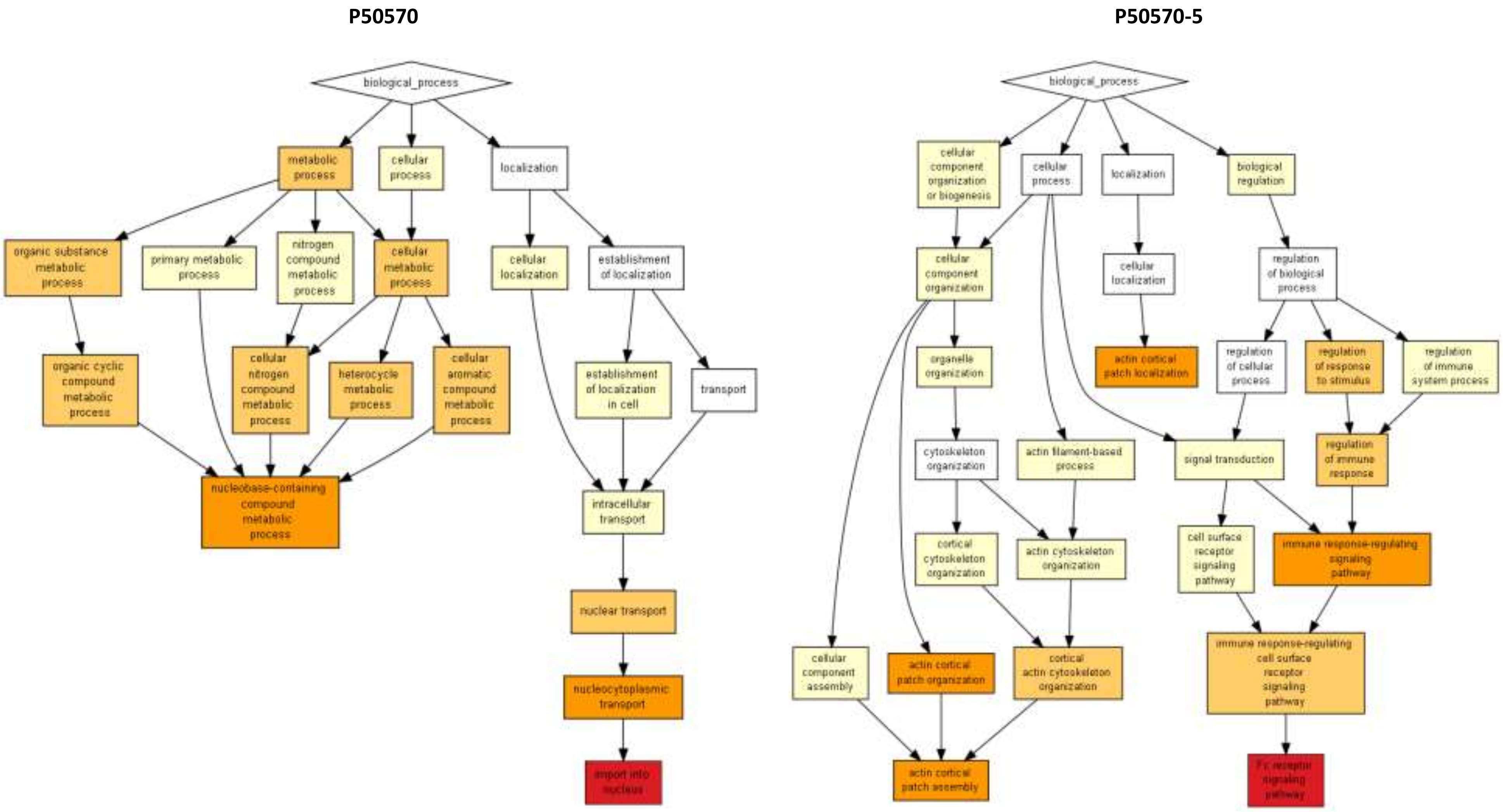

GO enrichment of two proteoforms (canonical – P50570 and splice form P50570-2) encoded by DNM2 based on analysis of their PPI. The color intensity encodes the significance of corresponding GO term enrichment.
