# Supplementary material for: Predicting Functions of Uncharacterized Human Proteins: From Canonical to Proteoforms
Source: Genes (Basel). 2020 Jun 21;11(6):677. doi: 10.3390/genes11060677 (PMC7350264; doi:10.3390/genes11060677)
Supplement: Supplementary file 1 [file genes-11-00677-s001.zip › SUPPLEMENTARY/Table S2.pdf]

Table S2. The list of 31 human genes encoding functionally different proteoforms

Gene	AC (canonical)	AC (splice form)	Similarity of PPIs (%)	
			our data	according to IntAct
HAX1	O00165	O00165-5	0	
PES1	O00541	O00541-2	0	
SNAP91	O60641	O60641-3	0	0*
LDHA	P00338	P00338-3	0	0.12
LYN	P07948	P07948-2	0.3	0.04*
PKM	P14618	P14618-2	0	0
YBX3	P16989	P16989-2	0	0.02*
ZSCAN20	P17040	P17040-4	0.24	0*
MAP4	P27816	P27816-5	0	
PSMB5	P28074	P28074-3	0	
ERP29	P30040	P30040-2	0	
ACTN2	P35609	P35609-2	0	
BSG	P35613	P35613-3	0	0*
NUDT1	P36639	P36639-3	0	
TAGLN2	P37802	P37802-2	0	
DNM2	P50570	P50570-5	0	
TCOF1	Q13428	Q13428-4	0	0*
SPTAN1	Q13813	Q13813-3	0.3	0.01*
GANAB	Q14697	Q14697-2	0.16	0
ELOC	Q15369	Q15369-2	0	
GLYR1	Q49A26	Q49A26-5	0	
CDKAL1	Q5VV42	Q5VV42-2	0.01	0.06*
KAT2A	Q92830	Q92830-2	0	0*
TTC17	Q96AE7	Q96AE7-2	0.31	
SERPINB12	Q96P63	Q96P63-2	0	
CCT7	Q99832	Q99832-4	0	
BRD8	Q9H0E9	Q9H0E9-2	0.17	0.18
SEPTIN11	Q9NVA2	Q9NVA2-2	0	0.04*
VPS29	Q9UBQ0	Q9UBQ0-2	0	0.18
VDAC3	Q9Y277	Q9Y277-2	0	0*

* at least for one protein the number of PPIs less than 10
