# Supplementary figures and images for: Predicting Functions of Uncharacterized Human Proteins: From Canonical to Proteoforms
Source: Genes (Basel). 2020 Jun 21;11(6):677. doi: 10.3390/genes11060677 (PMC7350264; doi:10.3390/genes11060677)

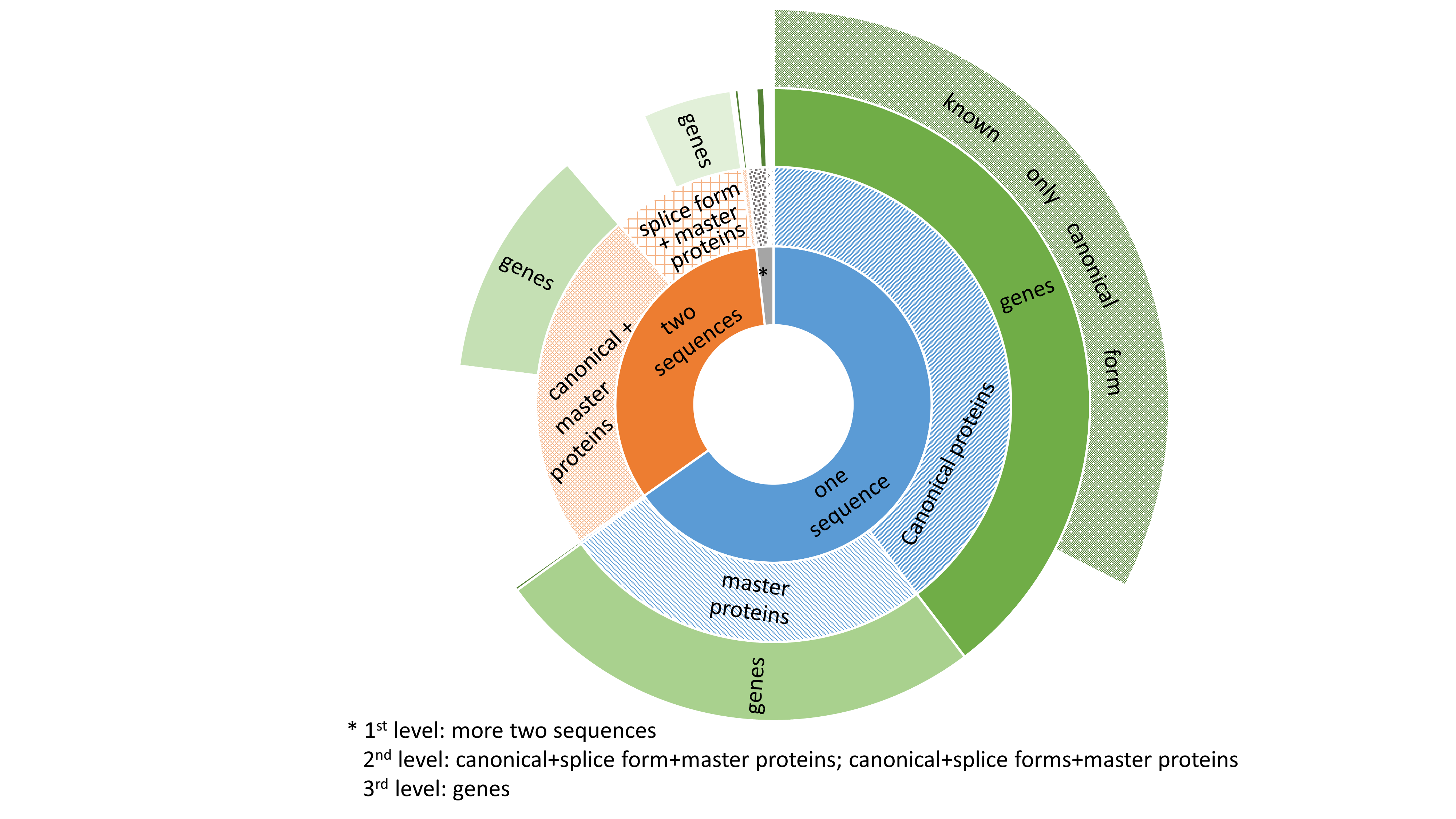

Supplement: Supplementary file 1 [file genes-11-00677-s001.zip › SUPPLEMENTARY/Figure S1.tif]

(a)

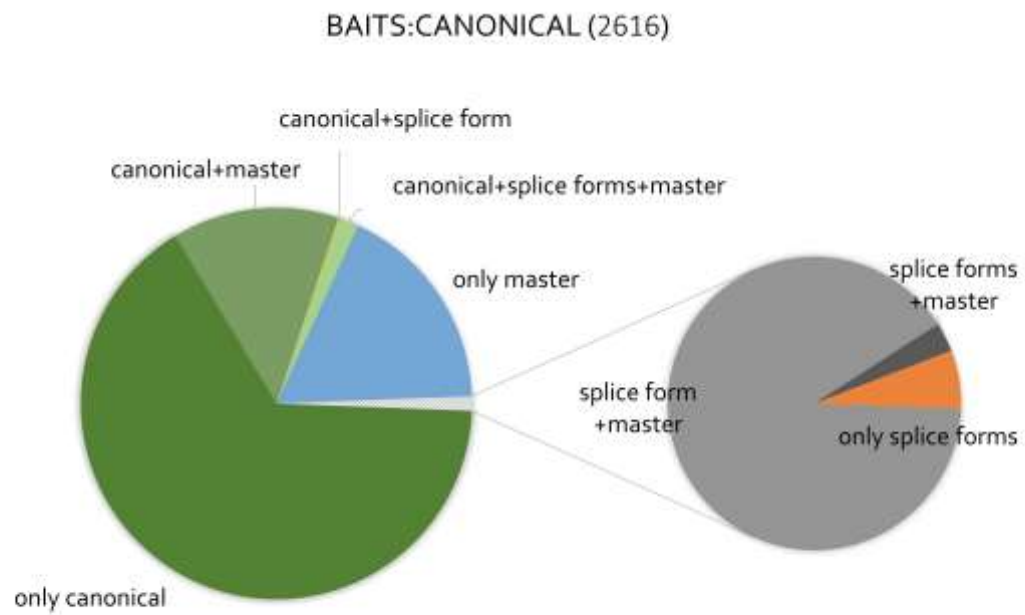

(b)

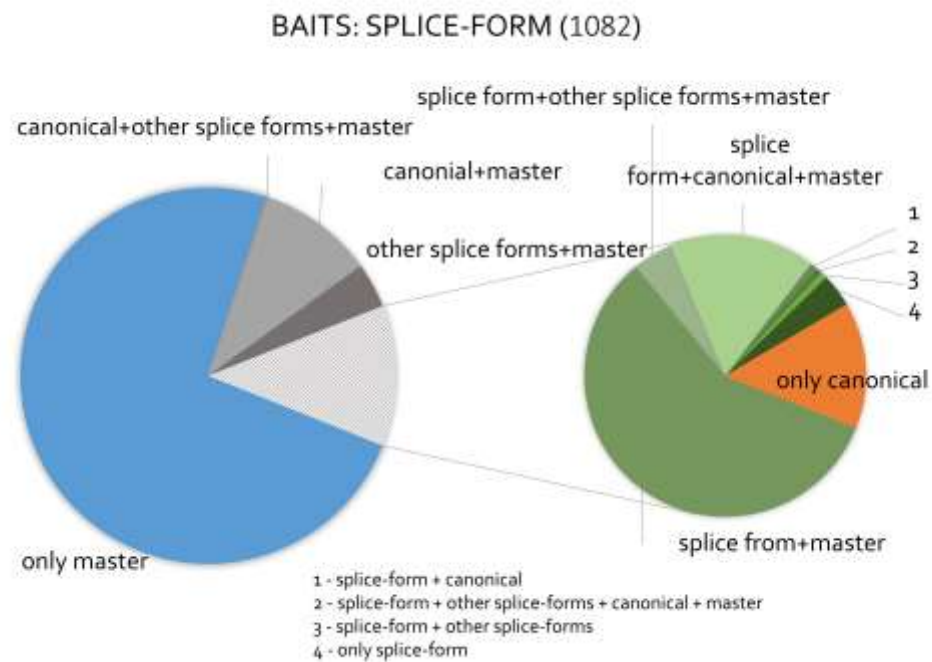

Supplement: Supplementary file 1 [file genes-11-00677-s001.zip › SUPPLEMENTARY/Figure S2a_b.pdf]

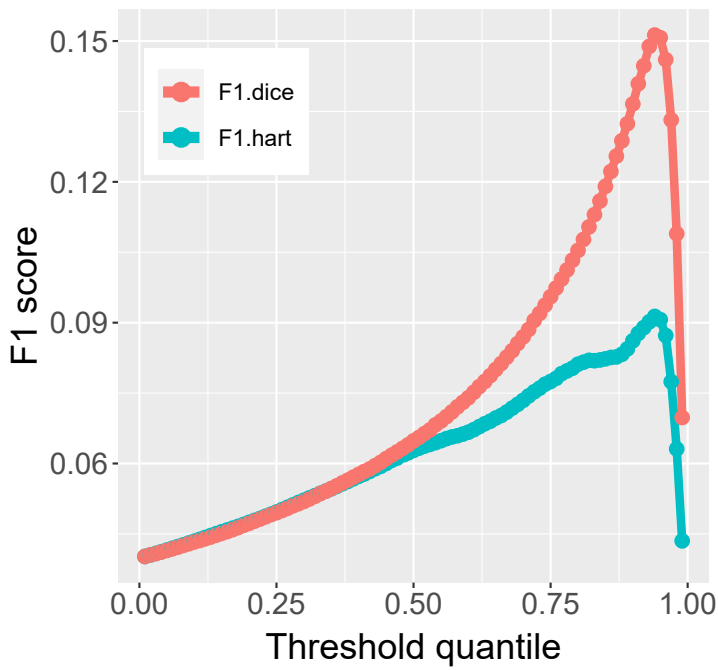

Supplement: Supplementary file 1 [file genes-11-00677-s001.zip › SUPPLEMENTARY/Figure S3.pdf]

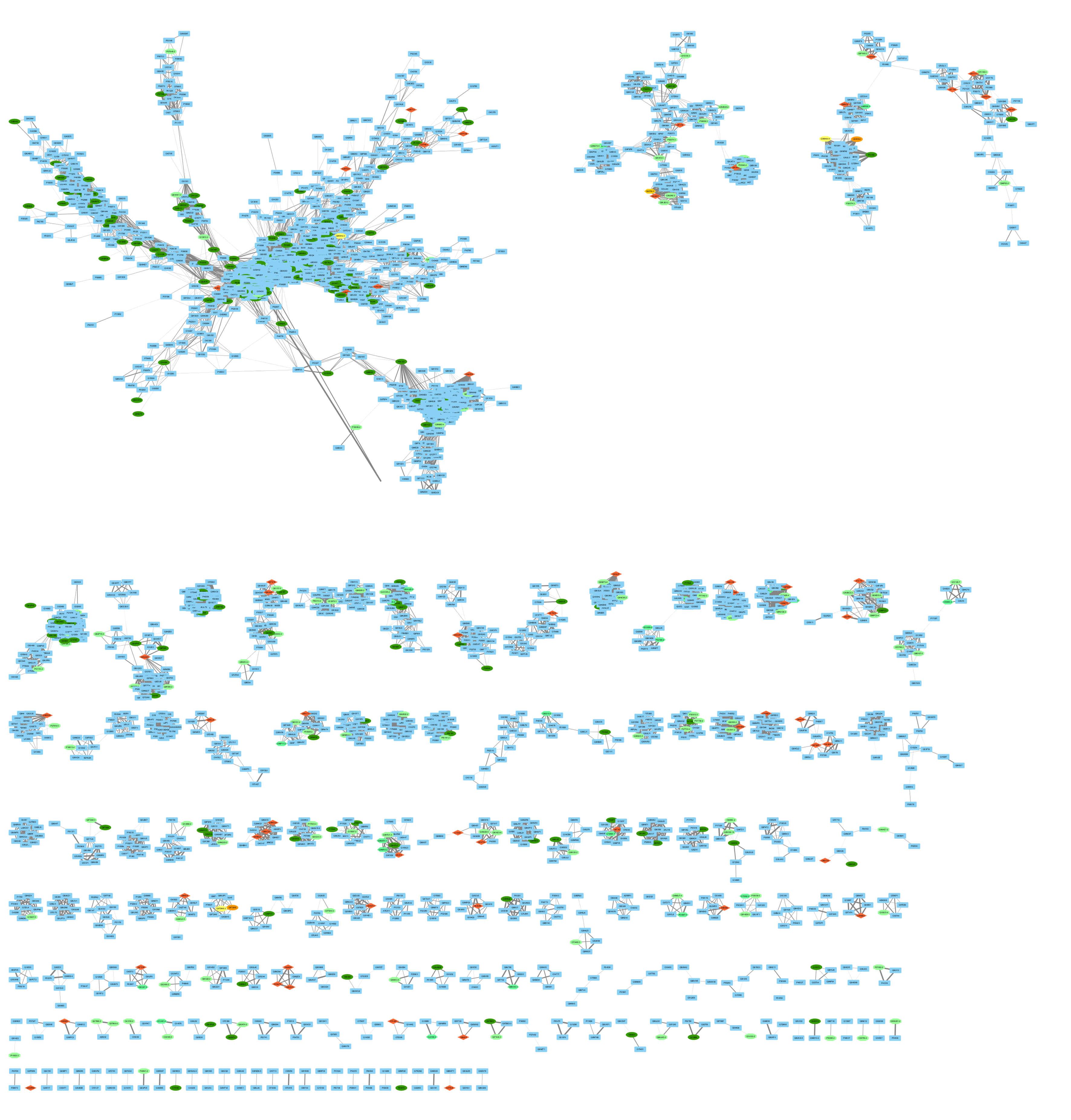

Supplement: Supplementary file 1 [file genes-11-00677-s001.zip › SUPPLEMENTARY/Figure S4.tiff]
